# YpfΦ: a filamentous phage acquired by *Yersinia pestis*

**DOI:** 10.3389/fmicb.2014.00701

**Published:** 2014-12-15

**Authors:** Anne Derbise, Elisabeth Carniel

**Affiliations:** Yersinia Research Unit, Department of Microbiology, Institut PasteurParis, France

**Keywords:** filamentous bacteriophage, plague, *Yersinia pestis*

## Abstract

*Yersinia pestis*, the plague bacillus, has an exceptional pathogenicity for humans. The plague bacillus emerged very recently (≈3,000 years ago) from the enteropathogen *Y. pseudotuberculosis.* Early after its emergence, *Y. pestis* became infected by a filamentous phage named YpfΦ. During the microevolution of the plague bacillus, the phage remained in the various lineages as an unstable extrachromosomal element. However, in the sub branch that caused the third plague pandemic, YpfΦ integrated itself into the bacterial chromosome to become a stable prophage. The genome of this phage has the same genetic organization as that of other filamentous phages such as the *Vibrio cholerae* CTXΦ phage, and shares high sequence identity with the CUS-1 filamentous phage of a high-virulence *Escherichia coli* K1 clone. In addition to genes involved in phage physiology, YpfΦ carries at each extremity of its genome two open reading frames with no predicted functions. This filamentous phage confers some selective properties to *Y. pestis* during the infectious process, which may explain why it was conserved during*Y. pestis* microevolution, despite its instability as an extrachromosomal element in most branches.

## INTRODUCTION

*Yersinia pestis*, one of the most dangerous bacterial pathogens of humans, is the causative agent of plague, a zoonotic disease transmitted from animals to humans by fleabites. After injection into the dermis, the bacteria migrate to the draining lymph node, where they cause the pathognomonic bubo. Bubonic plague, the most common clinical form, is fatal in less than a week in 40–70% of the patients if left untreated. Pneumonic plague, the second most common form of the disease, results from human-to-human transmission of the bacillus by aerosols, and is systematically and rapidly lethal if effective antibiotic therapy is not delivered before or at the onset of symptoms. Despite considerable progress in plague prevention and cure, this infection has not been eradicated. Natural plague foci still persist in numerous countries in Africa, Asia, and the Americas. Several genetic elements conferring virulence properties have been horizontally acquired by *Y. pestis*. This includes three plasmids: (i) pYV, which encodes a type III secretion system and toxins that subvert the defenses of the mammalian hosts, (ii) pFra, a large replicon which codes for a capsule that confers some resistance to the antibacterial activity of macrophages, and (iii) pPla, whose main product (Pla) is an important virulence factor that has protease and plasminogen activator activities. Another horizontally acquired element is the High Pathogenicity Island which allows *Y. pestis* to utilize the host iron and to cause septicemia. The most recently described mobile element is a filamentous phage, named YpfΦ that plays a role in the capacity of the plague bacillus to multiply and disseminate in mice (Derbise et al., 2007). The purpose of this review is to present the current knowledge about YpfΦ and to discuss its impact on the plague bacillus physiology.

## CHARACTERISTICS OF YpfΦ

**FIGURE 1:**
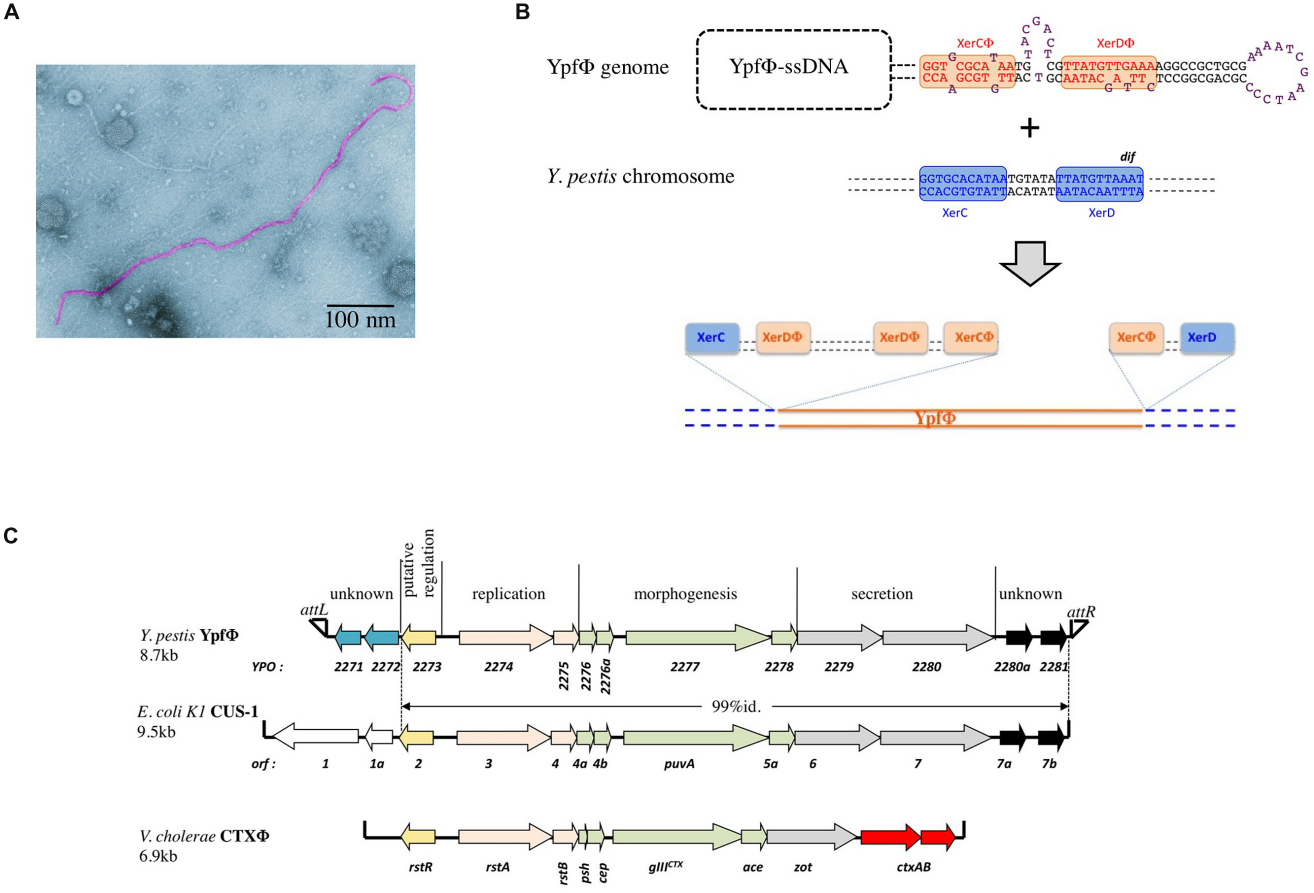
**(A)** Electronic micrograph of YpfΦ. **(B)** Schematic representation of the proposed XerC-dependent phage integration into the chromosomal *dif* site of *Yersinia pestis.* **(C)** YpfΦ, CUS-1 and CTX-1 genomic organization.

YpfΦ forms filamentous particles that are secreted by the *Y. pestis* cells into the culture supernatant without affecting bacterial growth or lyzing the cells. Low titers of phages are produced in standard *in vitro* growth conditions. YpfΦ has a filamentous virion 1,200 nm in length and 8 nm in diameter, which contains a circular positive single strand DNA molecule (**Figure 1A**). The phage has the capacity under laboratory conditions to infect the three pathogenic *Yersinia* species (*Y. pestis* and the enteropathogens *Y. pseudotuberculosis* and *Y. enterocolitica*). The phage infectivity rates, determined with an antibiotic tagged version of YpfΦ range from <2 × 10^-9^ to 10^-1^ depending on the isolates. *Y. pestis* strains are the most susceptible to a YpfΦ infection (99% of the strains), followed by *Y. enterocolitica* (50% of the strains), while only 30% of the *Y. pseudotuberculosis* strains are infected. The infectivity spectrum of YpfΦ is not restricted to *Yersinia* since *Escherichia coli* strains (TOP10, ECOR31) were also successfully transduced with YpfΦ.

No YpfΦ receptor on the bacterial surface has been identified yet. Deletion of genes encoding pili-like structures (*pilA*, *psaA*) that were potential candidate receptors did not affect the susceptibility of *Y. pestis* to YpfΦ (Chouikha et al., 2010). It could be possible that YpfΦ uses several receptor molecules at the bacterial surface, a hypothesis that is supported by the wide host range of this phage. Many filamentous phages use the TolQAR complex as a membrane receptor for entry into the recipient cell. Since TolQAR is highly conserved in Gram negative bacteria (Click and Webster, 1997; Heilpern and Waldor, 2000), it could serve as a secondary receptor for YpfΦ entry (Russel et al., 1988).

## ORIGIN AND DISTRIBUTION OF YpfΦ

**FIGURE 2:**
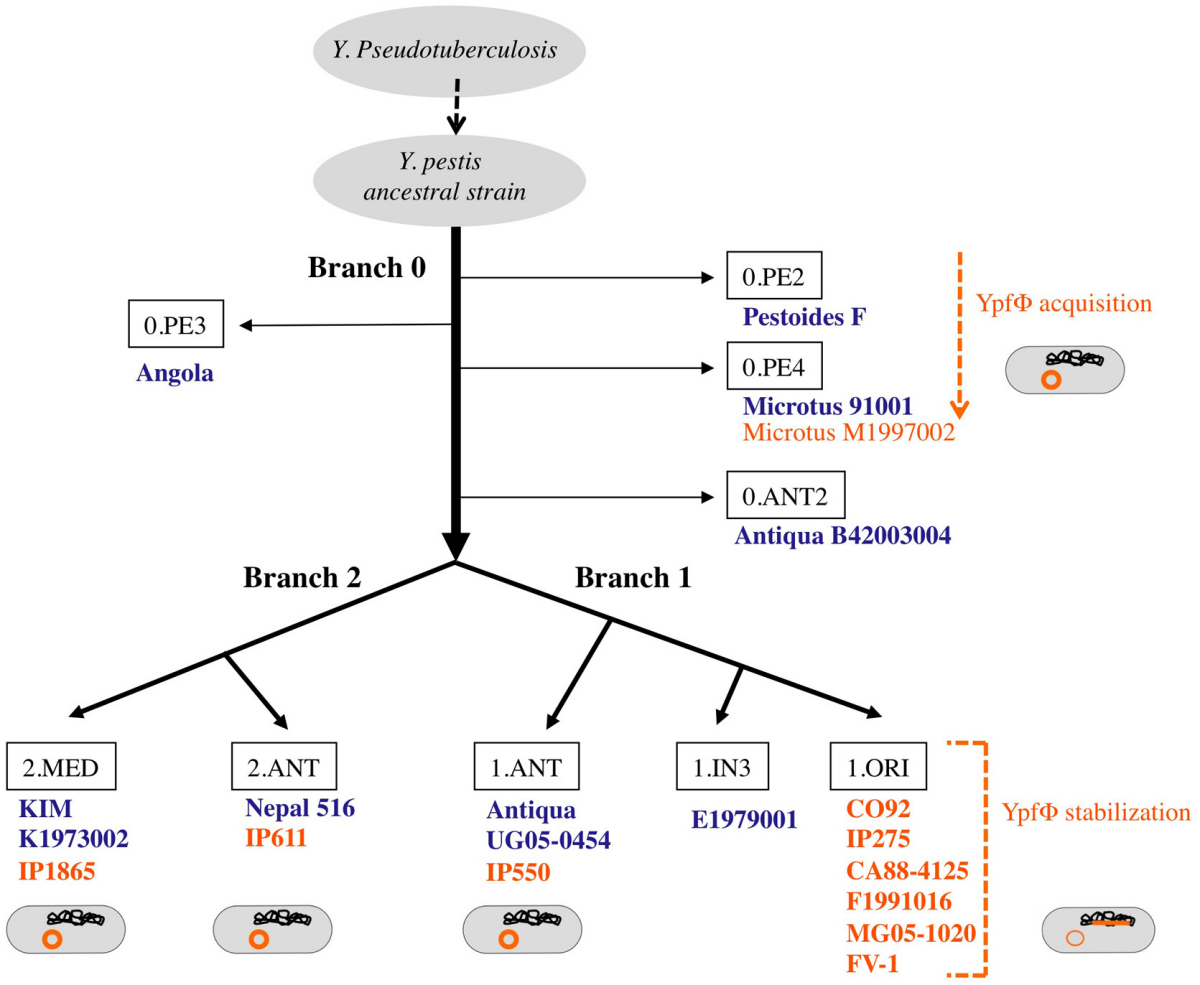
**Microevolution tree of *Y. pestis*.** Strains in which no YpfΦ was identified in the sequenced genome are indicated in blue. Strains from which a phage genome was detected by PCR and/or sequencing are indicated in orange. The location of the phage genome within the bacterial cell is schematized in orange.

*Yersinia pestis* is a clonal species that emerged recently from *Y. pseudotuberculosis*, a much less virulent bacterium (Achtman et al., 1999). Molecular phylogenetic studies combined with whole genome sequencing showed that after its divergence from *Y. pseudotuberculosis*, *Y. pestis* evolved along one branch (branch 0), before the split into two main branches (1 and 2) and several sub branches (**Figure 2**; Achtman et al., 2004). The YpfΦ prophage genome was initially identified in a comparative study of the genomes of one strain each of *Y. pestis* and *Y. pseudotuberculosis*. An analysis of an extensive set of isolates showed that the phage is systematically absent from *Y. pseudotuberculosis*. In *Y. pestis*, the phage is present in isolates from the three main phylogenetic branches, suggesting its acquisition early after *Y. pestis* emergence (Derbise et al., 2007). However, YpfΦ was not detected in all *Y. pestis* isolates. The phage is systematically present in strains from sub branch 1.ORI (**Figure 2**). This branch corresponds to the *Y. pestis* isolates that caused the third plague pandemic and that are found in most plague foci worldwide today. In the other sub branches YpfΦ is detected in some isolates only. Furthermore, only a portion of the bacterial cells that were found to be phage-positive harbors the phage genome (Derbise et al., 2007; Li et al., 2008). This indicates that YpfΦ is not stably maintained in the non-1.ORI branches and that the phage is easily lost upon *Y. pestis* subcultures *in vitro*. The capacity to stabilize the phage genome in the bacterial chromosome as a prophage is thus a property that was acquired late during *Y. pestis* evolution, and only in the third pandemic lineage.

## LOCATION OF YpfΦ GENOME IN THE BACTERIAL CELL

In sub branch 1.ORI, YpfΦ is present mostly as an integrated prophage although extrachromosomal forms are also detected. As observed for several other filamentous prophages (Gonzalez et al., 2002; Huber and Waldor, 2002) YpfΦ integrates its genome into the chromosomal *dif* site of the host bacterium. *dif* is a recombinational locus used by the XerCD recombinases of the bacterial host to resolve chromosome dimers (Das et al., 2013). YpfΦ insertion reconstitutes an intact *dif* site at the 3′ extremity of the prophage sequence (**Figure 1B**). Sequence analysis of the YpfΦ encapsidated genome indicates the presence of a potential pair of binding sites for XerC and XerD in inverted orientations (**Figure 1B**). Similarly to the *Vibrio cholerae* filamentous phage CTXΦ such structure may constitute a XerCD substrate for recombination with the bacterial *dif* site, leading to the integration of the phage genome into the host chromosome (**Figure 1B**; Val et al., 2005). In the *Y. pestis* chromosome, the YpfΦ genome forms tandem repeats of two to four copies. Although two copies is the predominant form, more copies can be detected in the same bacterial population, suggesting constant and dynamic rearrangements between tandem repeats. Variations in the number of tandem repeats may also result from continuous excision by homologous recombination of some copies and insertion of extrachromosomal phage genomes. Supporting this hypothesis is the recent demonstration that EndoIII, a DNA glycosylase/lyase, serves as a co-factor facilitating the integration of CTXΦ ssDNA into the *V. cholerae* chromosomal *dif* site and increasing the number of tandem repeats (Bischerour et al., 2012). Since *Y. pestis* harbors in its genome a homologue to EndoIII, this protein may promote multiple successive integration events leading to the generation of YpfΦ tandem repeats.

In the phage-positive *Y. pestis* strains that belong to phylogenetic branches other than 1.ORI, the phage genome is present as an extrachromosomal replicon only. The difference in location, as compared with the 1.ORI strains, is not due to differences in phage properties since the YpfΦ nucleotide sequence is 100% identical in all *Y. pestis* isolates (Derbise et al., 2007). The chromosomal *dif* site and machinery of phage integration are also identical in all *Y. pestis* strains. Moreover, when phage-negative *Y. pestis* strains are infected under laboratory conditions with YpfΦ the phage genome can be found integrated into their chromosome. This confirms that these strains do have a functional integration machinery. Why the non-1.ORI natural isolates of *Y. pestis* do not carry prophage forms of YpfΦ remains an unanswered question Epigenetic differences might be involved. For instance the integration machinery could be differently expressed in the non-1.ORI strains, thus affecting YpfΦ insertion efficiency.

When *Y. pseudotuberculosis* and *E. coli* strains are transduced with YpfΦ under laboratory conditions, the phage is almost exclusively extrachromosomal, while in *Y. enterocolitica* the phage is both inserted in the chromosome and in extrachromosomal forms. Since the XerCD recombinases responsible for filamentous phage integration at the *dif* site are 99 and 100% identical between *Y. pestis* and *Y. pseudotuberculosis,* and the EndoIII sequence is 100% identical, the difference in integration efficiency may be explained either by differences in other integration host factors or by a higher excision rate of the phage genome from the bacterial chromosome.

The presence of an endogenous YpfΦ confers partial protection against a superinfection by the same phage. This is reminiscent of the immunity observed with CTXΦ which is due to a repressor of the replication encoded by the phage (Kimsey and Waldor, 1998). However when the YpfΦ superinfection occurs, the incoming phage integrates preferentially between two integrated copies of the phage genomes rather than at the 3′ extremity of the tandem repeats, as seen for CTXΦ. Since the junction between two integrated copies of YpfΦ constitutes an imperfect *dif* site (Chouikha et al., 2010), this insertion site was not predicted by classical models of phage integration. The YpfΦ insertion may also result from homologous recombination between an extrachromosomal incoming DNA and the chromosomal resident copies.

## GENETIC ORGANIZATION OF YpfΦ

The YpfΦ genome is 8.7 kb long and comprises 13 open reading frames (ORFs) organized in two opposite transcriptional orientations, with an intergenic region believed to be involved in transcription initiation (**Figure 1C**). Its genetic organization resembles those of well characterized filamentous bacteriophages such as CTXΦ of *V. cholerae* (Davis and Waldor, 2003) and Ff (f1, fd, and M13) of *E. coli* (Model and Russel, 1988). Eight ORFs are organized in three modules involved in phage replication (YPO2274 and YPO2275), morphogenesis (YPO2276-YPO2278), and secretion (YPO2279 and YPO2280). The role of these functional modules was confirmed after disruption of genes predicted to be involved in morphogenesis (YPO2277), secretion (YPO2279), or replication (YPO2274), as each mutation abolished the production of phage particles (Chouikha et al., 2010).

Two additional ORFs (YPO2280a and YPO2281) of the YpfΦ prophage, located immediately adjacent to the *attR*, have no predictable functions. CUS-1, a very similar filamentous prophage of an *E. coli* K1 high-virulence strain is almost identical (99% nucleotide identity) over its 7.1 kb segment, covering, besides the YPO2280a and YPO2281 ORF homologs, all phage genes required for regulation, replication and assembly (**Figure 1C**; Gonzalez et al., 2002). The YPO2280a and YPO2281 homologs are absent from CTXΦ, which carries at this position two other ORFs, encoding the cholera toxin, the major virulence factor of *V. cholerae*.

The attL-adjacent segment of YpfΦ is composed of two ORFs (YPO2271 and YPO2272) that are absent from CTXΦ and that are replaced by two other unrelated ORFs (*orf1* and *orf1a*) in CUS-1 (**Figure 1C**). These two ORFs have no homologs in the databases and are therefore of unknown functions.

## REGULATION OF YpfΦ PRODUCTION

YpfΦ carries YPO2273, a gene homologous to RstR, which is a transcriptional repressor of CTXΦ. YPO2273 is located at the same position as *rstR* in the phage genome, thus suggesting that it might also regulate the bacteriophage replication. However its regulatory role awaits experimental demonstration. In addition, YpfΦ is regulated by the *Yersinia* global regulator RovA, which binds to the putative promoter regions of YPO2274. In the absence of RovA, transcription of the phage genes YPO2274 to YPO2279 is highly increased (Cathelyn et al., 2006). Whether RovA interferes with YPO2273 expression is not known.

## ROLE OF YpfΦ IN *Y. pestis* PATHOGENESIS

In *Y. pestis*, a slight increase in the LD_50_ of the YpfΦ deleted strain (≈sevenfold) was observed in the mouse experimental model of bubonic plague. Furthermore, *in vivo* competitive assays showed that the presence of the phage conferred some advantages to the host bacteria, allowing a better colonization of their mammalian host. Deletion of the prophage genomes from the bacterial chromosome had no impact on *Y. pestis* capacity to grow *in vitro*, to be taken up by fleas and to multiply in their gut. Therefore, YpfΦ is not a major virulence factor of *Y. pestis*, but seems to confer a higher fitness to its bacterial host during the infection of mammals. This is similar to the effect of closely related phage CUS-1 in *E. coli* O18:K1:H7 invasive extra intestinal clones. Decreased *in vivo* fitness of an *E. coli* K1 mutant in the CUS-1 prophage containing an interrupted *puvA* gene (encoding a virion protein required for binding to the host receptors; **Figure 1C**) suggests that CUS-1 plays a role in *E. coli* virulence (Gonzalez et al., 2001). Overall, in contrast to the filamentous phage CTXΦ, that is crucial for the pathogenicity of *V. cholerae*, YpfΦ and CUS-1 have moderate impact on bacterial fitness and pathogenicity. Nevertheless, the fact that episomal YpfΦ has been maintained in the different *Y. pestis* branches despite the observed high rate of the phage loss *in vitro*, suggests that the presence of this mobile element provides an overall selective advantage to the plague bacillus.

## CONCLUSION

The YpfΦ filamentous phage has been acquired by *Y. pestis* after its divergence from *Y. pseudotuberculosis*, first as an extrachromosomal replicon, and subsequently as a stable, integrated prophage in the 1-ORI branch, the contemporary pathogen and the cause of the last plague pandemic. Whether the stabilization of the phage genome in this branch participated in its current pandemic spread is not known. Another yet unanswered question is why YpfΦ is capable of integrating itself into the bacterial chromosome in this lineage and not in the other ones, despite identical site and machinery of integration. Finally, identifying the functions of the ORFs located at each of the termini of the YpfΦ prophage genome could bring important insights into the function of the phage.

## Conflict of Interest Statement

The authors declare that the research was conducted in the absence of any commercial or financial relationships that could be construed as a potential conflict of interest.
